# Comparative Physiological and Proteomic Analysis Reveals the Leaf Response to Cadmium-Induced Stress in Poplar (*Populus yunnanensis*)

**DOI:** 10.1371/journal.pone.0137396

**Published:** 2015-09-08

**Authors:** Yunqiang Yang, Xiong Li, Shihai Yang, Yanli Zhou, Chao Dong, Jian Ren, Xudong Sun, Yongping Yang

**Affiliations:** 1 Key Laboratory for Plant Diversity and Biogeography of East Asia, Kunming Institute of Botany, Chinese Academy of Science, Kunming, 650204, China; 2 Plant Germplasm and Genomics Center, Kunming Institute of Botany, Chinese Academy of Sciences, Kunming, 650201, China; 3 Institute of Tibetan Plateau Research at Kunming, Kunming Institute of Botany, Chinese Academy of Sciences, Kunming, 650201, China; 4 Key Laboratory of Tibetan Environment Changes and Land Surface Processes, Institute of Tibetan Plateau Research, Chinese Academy of Sciences, Beijing, 100085, China; 5 Department of Grassland Science, Yunnan Agricultural University, Kunming, 650201, China; 6 University of Chinese Academy of Sciences, Beijing, 100049, China; Henan Agricultural Univerisity, CHINA

## Abstract

Excess amounts of heavy metals are important environmental pollutants with significant ecological and nutritional effects. Cdmium (Cd) is of particular concern because of its widespread occurrence and high toxicity. We conducted physiological and proteomic analyses to improve our understanding of the responses of *Populus yunnanensis* to Cd stress. The plantlets experienced two apparent stages in their response to Cd stress. During the first stage, transiently induced defense-response molecules, photosynthesis- and energy-associated proteins, antioxidant enzymes and heat shock proteins (HSPs) accumulated to enhance protein stability and establish a new cellular homeostasis. This activity explains why plant photosynthetic capability during this period barely changed. During the second stage, a decline of ribulose-1, 5-bisphosphate carboxylase (RuBisCO) and HSP levels led to imbalance of the plant photosynthetic system. Additionally, the expression of Mitogen-activated protein kinase 3 (MPK3), Mitogen-activated protein kinase 6 (MPK6) and a homeobox-leucine zipper protein was higher in the second stage. Higher expression of caffeoyl-CoA O-methyltransferase (CCoAOMT) may regulate plant cell wall synthesis for greater Cd storage. These genes may be candidates for further research and use in genetic manipulation of poplar tolerance to Cd stress.

## Introduction

Heavy metals constitute an important and worrying form of environmental pollution primarily caused by the increased mining and industrial activities in the 19^th^ and early 20^th^ centuries [1–3]. Fifty-three of the ninety naturally occurring elements are heavy metals [4]. Among heavy metals, Cd is of particular concern because of its widespread occurrence and high toxicity. A problem in some agricultural soils in China is the uptake of Cd by rice (*Oryza sativa*) and other crops grown on the acidic red soils of southern China [5,6]. The concentration of Cd in these soils exceeds the World Health Organization’s recommendations (0.2 ppm) [7]. Forest soils are more susceptible to Cd because they are often acidic, poorly buffered and exposed to atmospheric heavy-metal pollution [6].

Cd enters plants through the root system and is quickly transported to the stem via the xylem and finally stored in leaf epidermal cells [8–10]. Cd stress in plant leaves harms photosynthesis and chlorophyll metabolism by disrupting the electron transport chain, aggregation of pigment protein complexes of the photosystems [8]. Cd also might affect directly chloroplast replication and cell division in the leaf to damage numerous cellular structures and membranes [9,10]. This impairs nutrient distribution and accelerates apoptosis and necrosis in leaves. Additionally, Cd can alter the uptake of minerals by plants through its effects on soil mineral availability, or by reducing the soil microbe population [1]. In general, Cd has been shown to interfere with the uptake, transport and use of Ca^2+^, Mg^2+^, P, K^+^ and water by plants [2]. It has been reported that Cd toxicity can indirectly lead to the production of reactive oxygen species (ROS), such as the superoxide anion (O_2_^−^), and hydrogen peroxide (H_2_O_2_) by interfering with the antioxidant defence system to cause oxidative damage to plants [11].

To reduce its damage, cadmium can be detoxified by phytochelatins, a class of glutathione-derived peptides containing repeating units of Glu and Cys that function by binding metal ions and transporting them to the vacuole [12,13]. Plant cells can also stimulate the activity of antioxidant enzymes such as superoxide dismutase (SOD; EC 1.15.1.1), catalase (CAT; EC 1.11.1.6), l-ascorbate peroxidase (APX; EC 1.11.1.11), glutathione reductase (GR; EC 1.6.4.2), dehydroascorbate reductase (DHAR; EC 1.8.5.1) and monodehydroascorbate reductase (MDHAR; EC 1.6.5.4) to neutralize the harmful effects of ROS release. Additionally, Cd induces genes encoding enzymes for the biosynthesis of phenylpropanoids, trehalose, polyamines and tryptophan, which have been identified as critical factors for resistance to various stresses [14,15]. Cd increases the expression of genes for dehydration-related aquaporin isoforms and pathogenesis-related proteins, and for proteins involved in remobilizing carbon from other energy sources [16,17].

Yunnan poplar (*Populus yunnanensis* Dode) is distributed in the high altitude areas of southwestern China. It plays an important role in forestry production, afforestation, and environmental conservation because of its fast growth rate, high biomass, and large populations. It is also one of the woody plants most commonly used in stress resistance studies because of its outstanding tolerance to harsh environmental conditions, including heavy metals, cold, drought, salinity, acid rain, elevated CO_2_, warming, drought and UV-B [18–21]. Previous studies have shown that stress-related proteins like heat shock proteins, proteinases and pathogenesis-related proteins are increased in poplar in response to Cd treatment [22]. However, because of the growing Cd content in the soils of southern China, more evidence of the effects of Cd on *P*. *yunnanensis* is still needed. To investigate the expression patterns of proteins in *P*. *yunnanensis* in response to cadmium, we performed comparative proteomic and physiological analyses. Our results will facilitate the elucidation of the Cd stress response of *P*. *yunnanensis* to Cd conditions in the acidic red soils of southern China, and provide more information on the underlying mechanism.

## Materials and Methods

### Ethics statement

Plant samples were collected in the Kunming suburbs, China, but not the authority responsible for a national park, protected area of land or private land. We declare that no specific permissions were required for these locations/activities. We confirm that the field studies did not involve endangered or protected species. There will be no conflict of ethics and interest.

### Poplar seedling growth and Cd treatment

Yunnan poplar (*Populus yunnanensis*) cuttings were obtained every 10 days as three repeat experiments during the March to April 2014 from male plants in the Kunming suburbs (N 25° 08′ 17.60″, E 102° 44′ 36.28″), Yunnan Province, Southeastern China. After surviving in the field for 30 d, rooting plants that averaged 7–9 leaves were transplanted into 1/4 Hoagland’s nutrient solution (pH 5.5) in growth chambers under controlled conditions (23°C and 16 h light/8 h dark, 200 μmol photons m^−2^ s^−1^ light intensity, relative humidity 75–80%). When plants reached the desired size (18–20 leaves), CdCl_2_ was added to the culture solution to a final concentration of 100 μM. The fifth leaf from the apex of each seedling was harvested for physiological and proteomic analysis after 0 (control), 4, 8 and 12 d of treatment.

### Chlorophyll fluorescence analyses

Chlorophyll fluorescence was measured with a Pulse-Amplitude-Modulation (PAM) Chlorophyll Fluorometer (Heinz-Walz GmbH, Effeltrich, Germany) as previously described [23]. Briefly, plants were dark-adapted for 30 min to measure the maximum quantum yield of photosystem II (PSII). Fv/Fm and electron transport rate (ETR) was recorded during a saturating photon pulse (4000 μmol m^−2^ s^−1^) using a whole leaf.

### Protein extraction

Leaves collected from treated *P*. *yunnanensis* were homogenized to a fine powder in a mortar with liquid nitrogen. Approximately 1.0 g of plant sample from a part of powder of 30 individuals with equivalent quantity was used for protein isolation. Total soluble proteins were extracted on ice in acetone containing 10% (w/v) trichloroacetic acid (TCA) and 0.07% (w/v) dithiothreitol (DTT). The samples were kept at −20°C for 4 h and centrifuged at 25,000 *g* for 30 min at 4°C. The pellets were washed with acetone containing 0.07% (w/v) DTT at −20°C for 30 min and then centrifuged (25,000 *g*, 20 min, 4°C) three times. Following a further centrifugation, each pellet was vacuum-dried and dissolved in urea buffer comprising 8-M urea, 20 mM DTT, 4% 3-[(3-Cholamidopropyl) dimethylammonio] propanesulfonate, and 2% ampholyte (pH 4–7). The solution was vigorously vortex-mixed for 1 h at room temperature, centrifuged at 20°C for 20 min at 25,000 *g*, and the supernatant was collected for 2-DE experiments.

### Two-dimensional electrophoresis and image analysis

A total of 900 μg of proteins extracted from each sample were used for 2-DE. First dimensional electrophoresis was performed using immobilized pH gradient (IPG) strips (pH 4–7, nonlinear, 17-cm ReadyStrip; Bio-Rad, Hercules, CA, USA) according to the manufacturer’s instructions. After isoelectric focusing, the IPG strips were equilibrated for 20 min in equilibration buffer (6-M urea, 20% w/v glycerol, 2% w/v SDS, and 50 mM Tris-HCl, pH 8.8) containing 1% w/v DTT, and then alkylated with 2.5% w/v iodoacetamide in equilibration buffer for 20 min. The strips were placed atop 12% w/v SDS-PAGE gels with no stacking gel. Twelve gels (three replicates for each treatment) ran simultaneously and electrophoresis was performed at 15°C and 3 W/gel for 1 h and then 15 W/gel using a PROTEAN II XL Cell (Bio-Rad). Gels were stained with Coomassie Brilliant Blue R-250, and then scanned using a GS-800 calibrated densitometer (Bio-Rad). The digitized protein spots on 2-D maps were quantitatively analyzed using PDQuest 2D analysis software (Bio-Rad) on the basis of their relative volumes. The optimized parameters were as follows: partial threshold, 4; saliency, 2.0; minimum area, 50. To verify the autodetected results, all spots were manually quantified by determining the ratio of the volume of each detected spot to the total volume of all spots on the gels.

### Protein identification for mass spectrometry analyses and functional classification

Protein spots corresponding to expression changes greater than 1.5 fold were manually excised from the gels, and in-gel trypsin digestion was conducted as described by Wang *et al*., with minor modifications [24]. Specifically, spots were destained with 50 mM NH_4_HCO_3_ for 1 h at 40°C, and reduced with 30% (v/v) acetonitrile (ACN) in 50 mM ammonium bicarbonate prior to DTT and iodoacetamide alkylation. The gels were then minced, air-dried, and rehydrated in 12.5 ng/μL sequencing-grade modified trypsin (Promega, Fitchburg, WI, USA) in 25 mM NH_4_HCO_3_ at 37°C for 16 h. The peptides were extracted three times with 0.1% (v/v) trifluoroacetic acid (TFA) and 50% (v/v) ACN, and MS analysis was conducted using a 4800 Plus Matrix-Assisted Laser Desorption/Ionization-Tandem Time of Flight (MALDI-TOF/TOF-MS) Proteomics Analyzer (Applied Biosystems, Bedford, MA, USA). MS acquisition and processing parameters were set to reflector positive mode and an 800–3500 Da acquisition mass range, respectively. The laser frequency was 50Hz, and 700 laser points were collected for each sample signal. For secondary MS analysis, four to six ion peaks with signal-to-noise ratios exceeding 100 were selected from each sample as precursors. TOF/TOF signal data for each precursor were then was accumulated with 2000 laser points. The primary and secondary mass spectra were transferred to Excel files and submitted to MASCOT (http://www.matrixscience.com) for protein identification, applying the following parameters: NCBI nr database, other green plants as the taxonomy parameter, no molecular weight restriction, one missed trypsin cleavage allowed, iodoacetamide-treated cysteine, oxidation of methionine, a peptide tolerance of 100 ppm, and an MS/MS tolerance of 0.25 kDa. Protein identifications were validated manually with at least three peptides matched, keratin contamination was removed, and the MOWSE threshold was set over 60 (*P* < 0.05). According to the MASCOT probability analysis, only significant hits were accepted for the identification of a protein sample. When peptides matched multiple proteins, the protein with the highest score was selected. The proteins were assigned Gene Ontology (GO) annotations by the Blast2GO software, and grouped according to their putative molecular functions [25].

### Antioxidant enzyme activity measurement

The activities of CAT, APX, GR and SOD were determined using previously described methods with minor modifications [26]. Plant sample from a part of powder of 30 individuals was grinded in 100 mM sodium phosphate buffer (pH 7.0) to extract SOD, and the soluble proteins were extracted by grinding the powder in 50 mM sodium phosphate buffer (pH 7.0) containing 1.0 mM ethylenediamine-tetraacetic acid (EDTA), 0.5% (v:v) Triton X-100, 1 mM ascorbate acid (AsA) and 1% (w/v) polyvinyl-pyrrolidone (PVPP) with a small amount of quartz sand on ice. The homogenate was centrifuged (Universal 200R, Hettich, Germany) at 12,000 *g* for 20 min at 4°C. For APX, CAT and GR activity measurement, the powder was homogenized in 50-mM Tris-HCl buffer (pH 7.0) containing 20% (v/v) glycerol, 1 mM AsA, 1 mM DTT, 1 mM EDTA, 1 mM GSH, 5 mM MgCl_2_ and 1% (w/v) PVPP. The homogenate was centrifuged at 12,000 *g* for 10 min at 4°C, and then the supernatant was centrifuged at 21,000 *g* for 15 min at 4°C. The resultant supernatant was collected for determination of antioxidant enzyme activities, and stored at −80°C for further analyses. CAT activity was determined by monitoring the decomposition of H_2_O_2_ at 240 nm. The reaction mixture contained 50 mM potassium phosphate buffer (pH 7.0) and enzyme extract in a 1 mL volume. The reaction was initiated by adding 10 mM H_2_O_2_. One unit of catalase is defined as the amount of enzyme that liberates half of the peroxide oxygen from 10 mM H_2_O_2_ solution in 60 s at 25°C. APX activity was determined by the decrease of absorbance at 290 nm. The reaction mixture contained 50 mM sodium phosphate buffer (pH 7.0), 1mM ascorbate, 2.5 mM H_2_O_2_ and a suitable volume of enzyme extract. GR activity was determined by the oxidation of NADPH at 340 nm. The reaction mixture was composed of 50 mM Tris-HCl buffer (pH 7.0), 5 mM MgCl_2_, 0.2 mM NADPH, 0.5 mM glutathione (oxidized form, GSSG) and an appropriate volume of enzyme extract in a 1 mL volume. The reaction was initiated by the addition of NADPH at 25°C. One unit of SOD is defined as the amount of enzyme that causes a 50% decrease in the SOD inhibitable NBT reduction. The reaction mixture (3 mL) was composed of 50 mM sodium phosphate buffer (pH 7.8), 13 mM methionine, 75 μM nitroblue tetrazolium (NBT), 16.7 μM riboflavin and an appropriate volume of enzyme extract. The reaction was initiated by light illumination.

### *In situ* H_2_O_2_, O_2_^−^ and malondialdehyde (MDA) detection

The *in situ* detection of H_2_O_2_ and O_2_^−^ was performed using a previously reported method [23]. H_2_O_2_ was monitored with 1mg/mL of diaminobenzidine. O_2_^−^ in the leaves was detected by NBT reduction at specific time points. The MDA content was determined as described by Duan *et al*. [27]. Approximately 0.5 g of fresh leaf tissue was homogenized in 10 mL of 10% TCA and centrifuged at 12,000 *g* for 10 min. Then, 2 mL of 0.6% thiobarbituric acid in 10% TCA was added to a 2 mL aliquot of the supernatant. The mixture was heated in boiling water for 30 min and then quickly cooled in an ice bath. After centrifugation at 10,000 *g* for 10 min, the absorbance of the supernatant at 450, 532, and 600 nm was determined. The MDA concentration was estimated using the formula: C (nmol/mL) = 6.45 (A_532_ − A_600_) − 0.56A_450_. The MDA concentration was expressed as n mol g^−1^ FW.

### Western blotting

Proteins were extracted as described above, and 20 μg of proteins were separated by SDS-PAGE using 12% (w/v) polyacrylamide slab gels. Electrophoresis was performed at 15°C and 80V for 30 min and then 120V using a PROTEAN® II XL Cell. Following electrophoresis, the proteins were electrotransferred to polyvinylidene difluoride (PVDF) membranes at 20 V for 25 min using a Trans-Blot SD (Bio-Rad). After transfer, the membranes were blocked in 5% dried milk for 1 h at room temperature (23–25°C) and incubated with the primary antibody, which was diluted to 1:3000 for anti-CCoAOMT, 1:1000 for anti-HSP70, 1:3000 for anti-HSP18.2, and 1:3000 for anti-MPK3 and anti-MPK6, at room temperature for 1 h, and with horseradish peroxidase (HPR)-conjugated secondary antibody for 1 h at room temperature. The antibodies against plant were obtained from Agrisera (Agrisera, Vannas, Sweden). The chemiluminescence signals were detected using an ECL kit (GE, Evansville, IN, USA).

## Results

### Photosynthesis changes during Cd treatment

**Fig 1:**
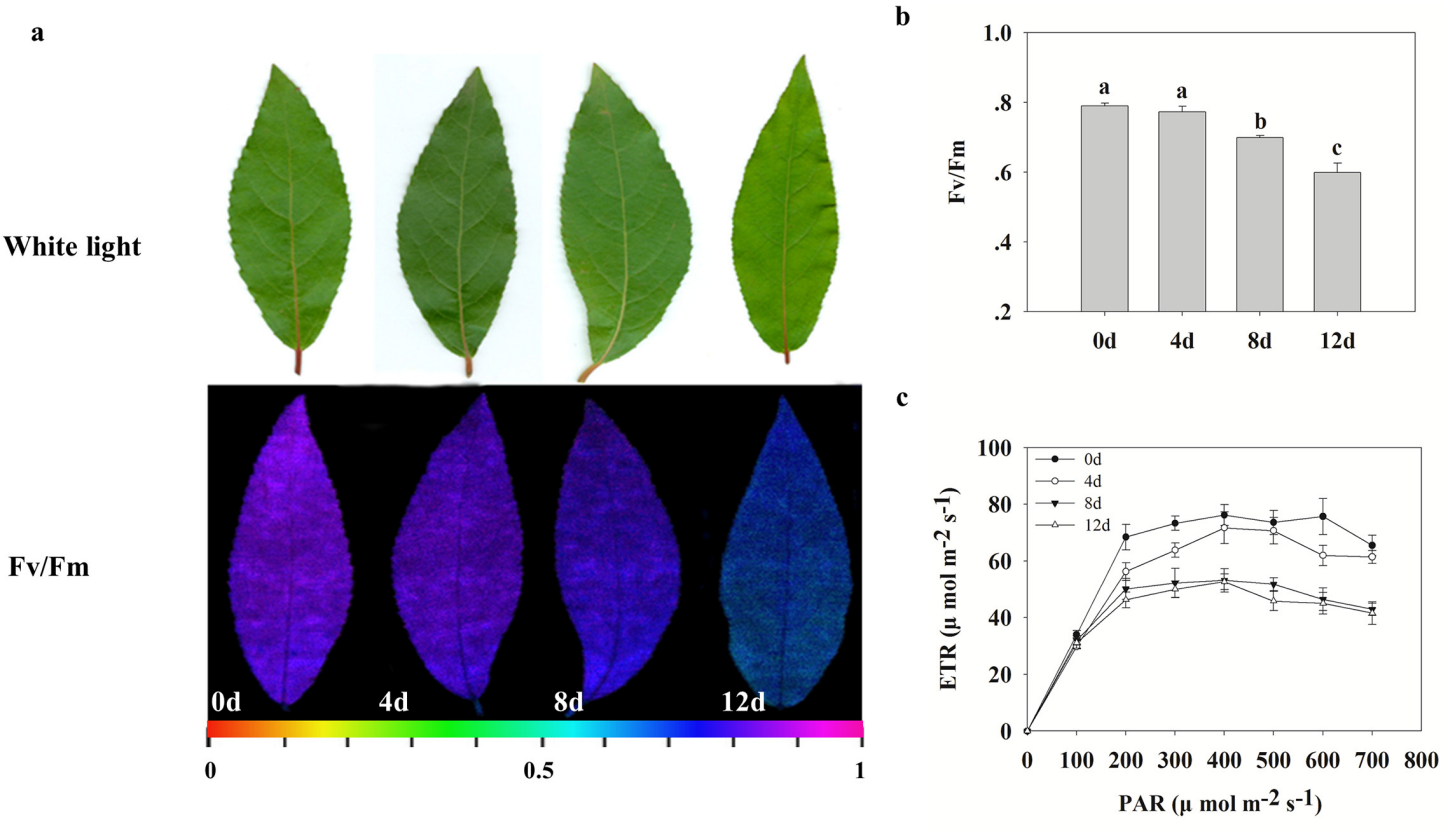
Effects of Cd on photosynthetic capability of leaves of *Populus yunnanensis* (a). The effects of Cd treatment on the Fv/Fm ratio (b) and ETR (c) in leaves after 0, 4, 8 and 12 d of treatment. Images were obtained (up), and photosynthetic capabilities were recorded by Fv/Fm imaging using a PAM chlorophyll fluorometer (down) at the indicated times. Images captured under white light were used as controls. The pseudocolor code depicted at the bottom of the images ranges from 0 (red) to 1.0 (purple). Values reflect means ± SEs of at three independent experiments (*n* = 30/experiment). Different symbols above the bars indicate significant differences (Tukey’s test, *P* < 0.05).

The Fv/Fm ratio and ETR in PSII can indicate the photosynthetic capacity of a plant. As shown in Fig 1a, from false-color images, changes in photosynthetic activity could be readily discerned. The highest negative effects occurred after 12 d of Cd treatment, whereas these parameters were not significant after 4 d (Fig 1b). Estimation of the PSII maximum efficiency by fluorescence images of Fv/Fm indicated that Cd reduced the Fv/Fm values of the samples (Fig 1a and 1b). The Fv/Fm values of samples treated for 8 and 12 d were lower, respectively, compared with the controls (0 d) (*P* < 0.05). Additionally, Cd decreased the ETR after 8 and 12 d of treatment (Fig 1c). When plants were stressed by Cd, ETR descended more rapidly and to greater degrees from 4 h to 8 h than from 0 h to 4 h. These results suggest the Cd decreases the photosynthetic activity of *P*. *yunnanensis* with the prolongation of Cd stress time, especially after 4 days of treatment.

### Dynamic changes of protein level Cd treatment

**Fig 2:**
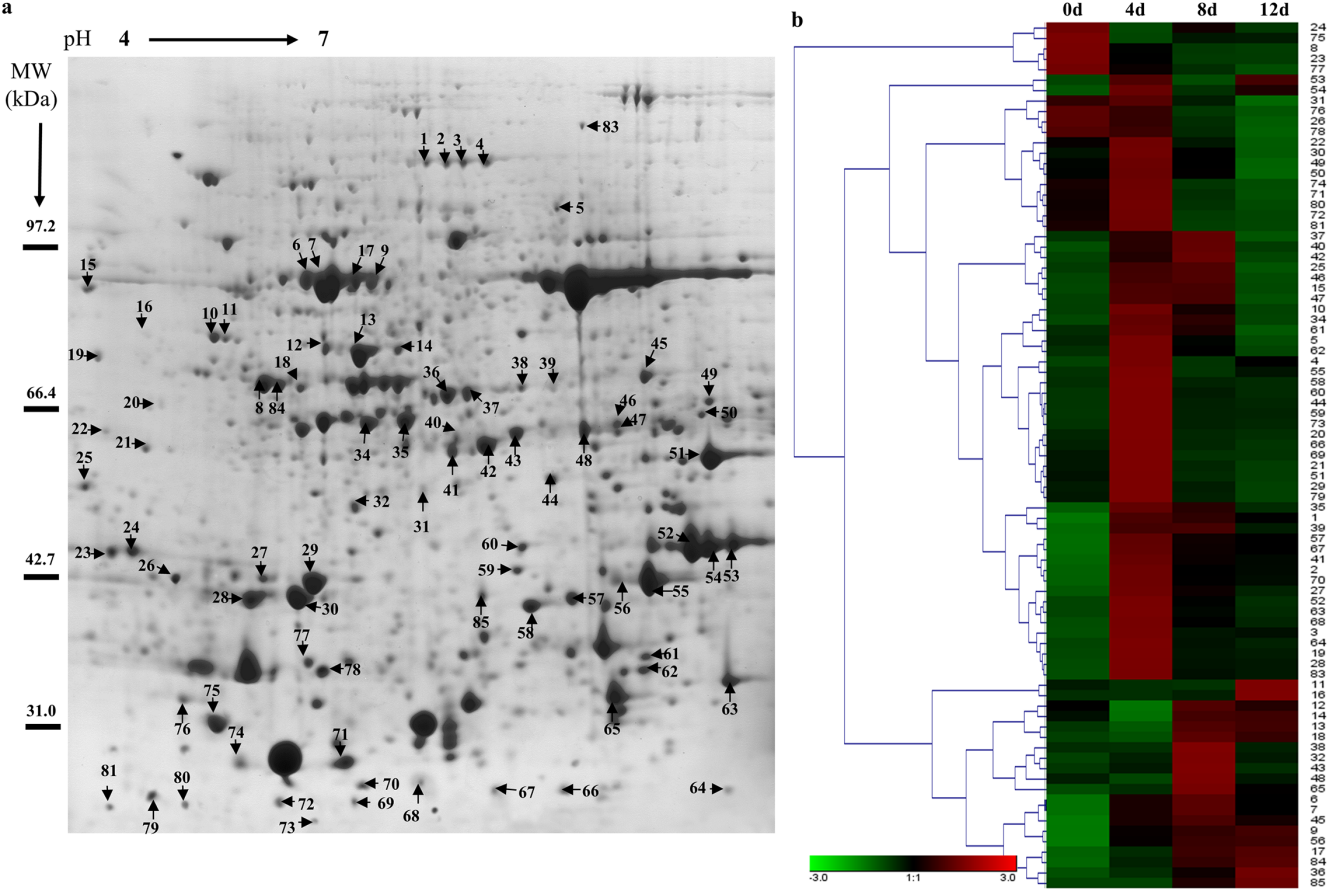
Protein expression patterns in the total proteins of poplar leaves exposed to Cd. 2-DE maps showing protein profiles of poplar leaves untreated (a). Proteins were extracted from leaves and separated by isoelectric focusing on an IPG strip of pH 4–7 (left–right), followed by SDS-PAGE on a 12% gel. (b) Hierarchical cluster analysis based on protein expression levels was performed with Genesis 1.7 software. Colors correspond to the log-transformed values of protein as shown in the bar at the bottom of the figure.

**Fig 3:**
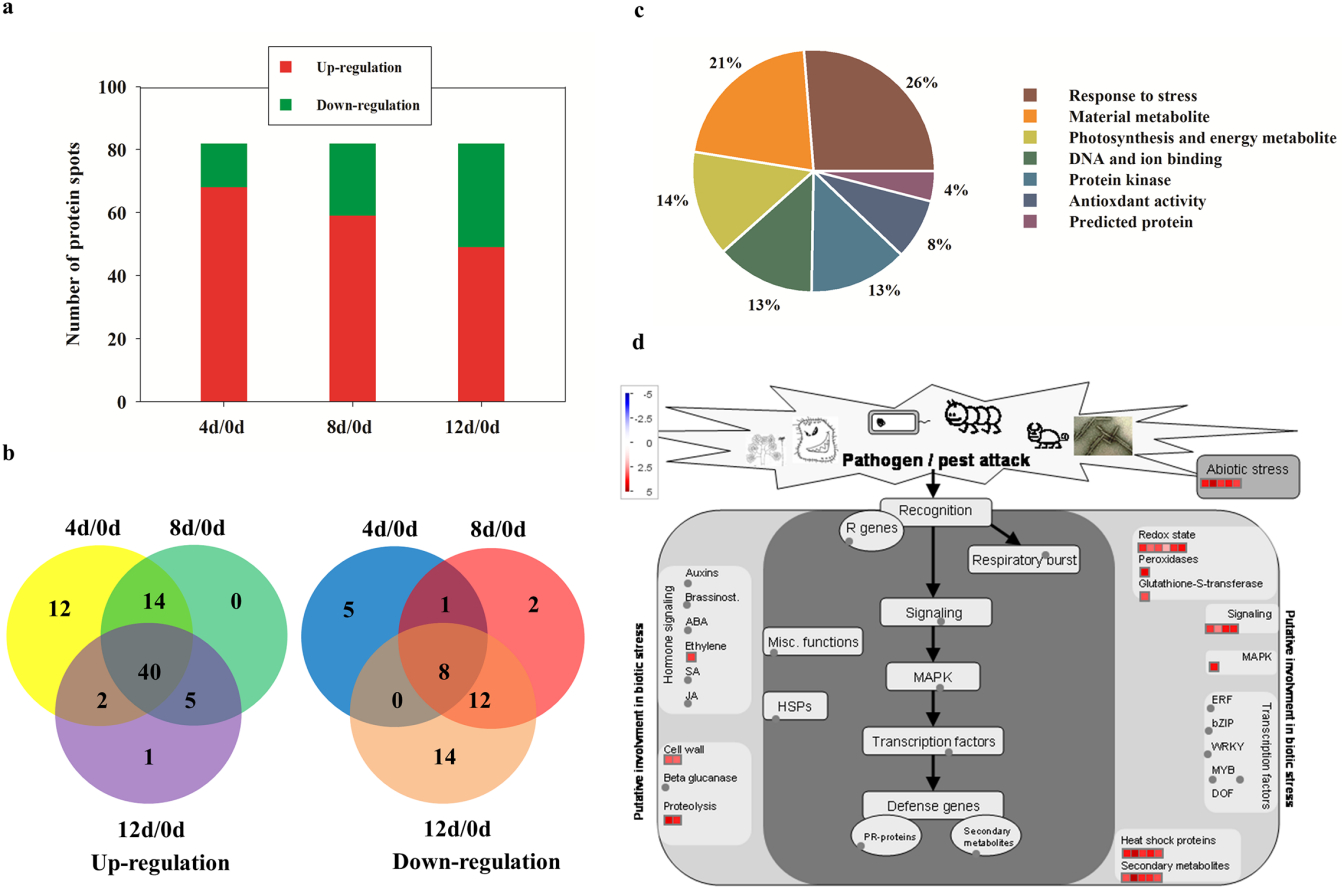
Results of comparative proteomics analyses of different treatments. (a) Number of identified proteins showing changes in expression at each treatment time. (b) Venn diagram showing the overlap of up- and down-regulated proteins among 4 d/0 d, 8 d/0 d, and 12 d/0 d. (c) Functional classification of differentially expressed proteins in poplar leaves after Cd treatment. (b) Proteins involved in biotic stress according to MapMan in leaves after 4 d of treatment. Details of the mapped proteins were shown in the S2 Table. Positive fold change values (red) indicate up-regulation, whereas negative fold change values (blue) indicate down-regulation. Gray circle mean the protein that is not mapped in the pathway.

To evaluate the expression patterns of proteins in poplar cells following Cd treatment, the total proteins of leaves sampled at 0, 4, 8 and 12 d were extracted and separated by 2-DE (Fig 2a and S1 Fig). More than 600 protein spots were reproducibly detected within each treatment. Eighty-three differentially expressed proteins (at least 1.5-fold, P<0.05) were detected using PDQuest 7.1 (Bio-Rad), and the expression levels of proteins in different treatments were shown and analyzed by Genesis 1.7 software (Fig 2b). Fig 3a shows the protein changes with cadmium treatment at different time intervals. We found that the numbers of up-regulated proteins decreased and down-regulated proteins increased after prolonged cadmium treatment. Forty proteins were found in the intersection of up-regulated proteins and eight proteins were found in the intersection of down-regulated proteins among 4 d/0 d, 8 d/0 d and 12 d/0 d (Fig 3b). These results demonstrated that some proteins expression levels first increased and then decreased in *P*. *yunnanensis* in response to cadmium stress, such as spots 74, 62, 79, and 10 (Table 1 and S1 Fig).

**Table 1:** Proteins identified by MALDI-TOF/TOF analysis of differentially expressed proteins spots.

Spots No.	NCBI accession No.^a^	Protein name	Theo. Mw/PI^b^	Exp. Mw/pI^c^	Score ^d^	SC ^e^	Ratio ^f^		
							4d/0d	8d/0d	12d/0d
Response to stress									
64	GI:224071519	Heat shock protein 22	23.91/6.35	23.36/6.74	107	22.88	1.41	1.11	1.07
58	GI:566213236	14-3-3 protein	29.66/4.75	40.41/6.16	78	19.08	1.53	1.03	1.01
74	GI:566189392	Heat shock protein 20	27.26/8.91	27.24/4.93	95	15	1.49	0.55	0.41
67	GI:224103619	Caffeoyl-CoA O-methyltransferase	27.81/5.02	33.23/4.72	168	14.17	1.88	1.52	1.46
81	GI:566220729	Gamma-thionin family	8.13/8.98	19.7/4.34	132	35.14	1.43	0.55	0.51
53	GI:224113841	Aminocyclopropane carboxylate oxidase	36.26/5.15	45.57/6.81	163	17.61	1.9	0.97	1.84
10	GI:224056837	Heat shock protein 90	80.02/4.95	76.26/4.88	92	8.58	1.57	1.28	0.99
12	GI:566168226	Stromal 70 kDa heat shock-related	75.21/5.35	76.1/5.27	122	6.24	0.49	1.34	1.18
31	GI:224105301	Latex plastidic aldolase-like protein	42.88/7.56	53.19/5.37	171	13.38	1.1	0.73	0.75
55	GI:224078584	Glyoxalase I homolog protein	33.15/5.19	43.49/6.48	99	13.94	2.19	0.99	1.21
41	GI:224065571	Succinyl-CoA ligase beta-chain protein	45.27/6.63	54.7/5.72	89	12.11	2.37	1.75	1.62
28	GI:224114557	Malate dehydrogenase	35.59/8.71	44.20/5.08	108	14.09	1.56	1.16	1.15
32	GI:566156447	No apical meristem (NAM) protein	46.10/8.76	57.50/5.45	107	10.53	1.08	1.5	1.1
23	GI:224094919	Plastidic aldolase	42.75/6.85	51.46/4.5	91	11.36	0.38	0.07	0.07
54	GI:231757	Caffeic acid 3-O-methyltransferase 1	39.73/5.48	45.73/6.72	185	12.36	1.91	1.17	1.55
30	GI:566242067	O-methyltransferase	39.81/5.28	45.73/5.17	83	13.45	1.64	1.09	0.65
14	GI:224098390	Heat shock protein 70	71.27/5.14	74.95/5.52	82	9.1	0.63	1.32	1.3
62	GI:224056110	Caffeoyl-CoA O-methyltransferase	27.95/5.33	33.30/6.46	96	14.98	2.01	1.29	0.86
72	Gi:147225064	23.1kDa heat-shock protein	23.24/5.04	23.14/5.04	109	16.11	1.47	0.61	0.56
3	GI:566169279	NBS-LRR resistance gene-like protein ARGH34	105.92/6.59	108.98/5.87	564	7.57	2.73	1.5	1.55
4	GI:566152761	RNA recognition motif	104.83/7.08	108.98/5.94	114	5.91	1.64	1.05	1.23
29	GI:224099381	Adenylate isopentenyltransferase	40.86/8.73	46.64/5.2	79	13.81	1.96	0.98	0.76
Material metabolite									
40	GI:566209075	Sucrose-phosphatase	47.85/5.76	58.49/5.64	98	10.12	1.57	1.84	1.09
21	GI:224102579	Glutamine synthetase	47.75/6.48	57.85/4.92	176	10.65	2.23	0.87	0.68
8	GI:566182944	Terpene synthase	88.79/5.73	85.03/5.37	92	8.57	0.5	0.29	0.27
6	GI:313770759	Sucrose synthase 1	92.49/6.23	86.15/5.21	136	7.01	2.32	2.92	2.06
20	GI:566159976	Tyrosine decarboxylase	53.89/5.71	63.61/4.93	105	11.48	1.47	0.99	0.97
16	GI:224137750	L-GALACTONO-1 protein	68.70/8.91	70.55/4.63	167	10.78	0.97	1.37	5.45
51	GI:224130888	S-adenosylmethionine synthetase 1 family protein	43.21/5.68	54.07/6.72	213	15.05	1.92	0.84	0.72
75	GI:224059488	Triosephosphate isomerase	27.49/7.64	31.28/4.90	92	11.76	0.04	0.27	0.25
52	GI:224107975	Homocysteine S-methyltransferase 3	36.73/5.54	45.57/6.67	267	15.38	3.78	1.96	1.33
27	GI:566205887	Glutamine synthetase protein	39.21/5.81	45.65/5.06	263	13.48	1.6	1.3	1.16
39	GI:566170187	2-isopropylmalate synthase	63.95/5.80	68.14/6.00	136	9.44	3.37	3.42	2.09
24	GI:566201067	Pyridoxal-phosphate dependent enzyme	42.71/9.05	50.57/4.58	95	14.18	0.08	0.53	0.17
36	GI:224140859	Glucose-6-phosphate 1-dehydrogenase	59.16/6.27	66.99/5.70	94	12.26	1.16	1.42	1.85
9	GI:224063766	Transketolase	80.58/6.29	85.03/5.43	120	10.35	1.65	1.86	1.95
44	GI:566209994	Putative diaminopimelate epimerase	42.82/6.19	52.55/6.09	93	12.34	2.27	1.15	1.11
26	GI:224115588	Epsilon2-COP protein	32.18/5.16	42.50/6.00	95	15.51	0.83	0.41	0.21
78	GI:224101549	Enoyl-CoA hydratase/isomerase protein	28.98/9.40	36.28/5.25	102	18.49	0.89	0.43	0.17
Photosynthesis and energy metabolite									
70	GI:224078826	Oxygen evolving enhancer protein 3	24.90/9.60	25.53/5.45	84	13.25	1.69	1.33	1.28
37	GI:566190494	H+-transporting two-sector ATPase family protein	59.81/5.96	67.53/5.78	218	7.71	2.24	2.99	0.56
38	GI:224099437	H+-transporting two-sector ATPase	59.97/5.91	68.04/5.93	84	8.94	0.99	1.6	1.04
66	GI:566192331	Thioredoxin-like 8	22.57/8.93	20.80/5.85	91	15.23	1.24	0.99	0.97
79	GI:283558285	Ribulose-1,5-bisphosphate carboxylase/oxygenase large subunit	16.31/5.46	20.22/4.64	86	23.13	1.51	0.97	0.85
68	GI:566171611	Ribulose bisphosphate carboxylase small chain 1A	20.39/8.65	20.47/5.36	120	19.34	1.41	1.16	1.09
63	GI:224083006	Chlorophyll a-b binding protein 2	28.09/5.29	34.02/6.79	85	13.26	1.73	1.23	1.1
35	GI:566192956	Coproporphyrinogen III oxidase	53.69/9.49	63.34/5.53	113	8.84	1.71	1.5	1.19
43	GI:224086078	Glyceraldehyde-3-phosphate dehydrogenase	48.17/6.76	58.61/5.99	132	9.73	1.3	2.86	1.1
60	GI:566180403	Chain A protein	40.47/8.71	46.20/5.97	107	12.67	1.99	1.14	1.06
34	GI:110227064	ATP synthase CF1 alpha chain	55.32/5.20	63.8/5.40	210	8.88	2.49	2.01	1.02
49	GI:566182581	Ribulose bisphosphate carboxylase/oxygenase activase	51.94/5.26	61.55/6.61	146	8.42	1.55	1.03	0.55
DNA and ion binding									
77	GI:224129938	Beta-expansin 4 precursor	29.03/5.82	37.26/5.22	105	15.33	0.52	0.25	0.1
13	GI:566212846	FtsH protease	73.18/5.67	75.11/5.41	176	7.55	0.9	1.36	1.32
5	GI:566157986	Zinc finger protein	100.79/6.48	100.38/6.08	186	5.57	2.25	1.26	0.68
73	GI:566175689	Calcium-binding protein	21.82/4.66	20.79/5.14	103	19.19	2.12	1.11	1.08
59	GI:224066943	Annexin 1 protein	35.97/6.15	44.32/5.95	190	13.92	2.39	1.16	1.14
19	GI:224124888	Ent-kaurenoic acid oxidase	56.98/8.99	65.61/4.58	165	9.13	2.06	1.27	1.24
80	GI:75330796	Putative calcium-binding protein	17.18/3.99	20.26/4.53	176	20.52	1.34	0.78	0.7
18	GI:566156245	Histone acetyltransferase	61.99/6.11	68.11/5.22	118	8.63	0.87	2.19	1.91
65	GI:224116496	MADS-box protein	27.27/9.50	30.69/6.32	104	14.11	1.23	2.46	1.59
42	GI:566167194	Glycerate dehydrogenase	48.80/6.11	58.88/5.86	89	8.97	1.77	2.32	0.97
45	GI:1346485	NADP-dependent malic enzyme	65.18/6.32	68.64/6.29	70	7.1	3.91	4.86	3.83
Antioxdant activity									
1	GI:566169059	Cellulose synthase 6	122.48/6.65	109.29/5.65	104	5.81	2.34	2.15	1.81
48	GI:566173508	Catalase 2	48.76/6.21	58.88/6.28	99	11.71	0.79	1.82	1.03
69	GI:566159172	Glutathione S-transferase	23.21/6.23	22.59/5.32	176	19.25	1.78	0.86	0.85
56	GI:224087140	Peroxidase	34.94/5.98	43.52/6.35	89	15.58	2.2	2.67	2.72
61	GI:566183212	Peroxiredoxin	28.30/7.15	34.97/6.46	168	14.45	1.54	1.28	0.84
22	GI:566175737	Monodehydroascorbate reductase	47.050/6.51	57.55/4.56	178	10.6	1.73	0.79	0.32
57	GI:566170791	L-ascorbate peroxidase	31.51/7.06	40.62/4.54	125	16.72	3.62	2.6	2.4
Protein kinase									
2	GI:566188866	Pyruvate phosphate dikinase	106.60/5.57	108.98/5.73	93	6.89	4.1	2.46	2.31
15	GI:566169617	NIMA-related protein kinase	69.60/9.51	72.7/4.35	82	6.94	2.16	2.12	0.98
17	GI:566196799	Mitogen-activated protein kinase	66.87/6.17	69.37/4.98	101	9.83	1.6	2.72	2.96
71	GI:224121954	Adenylate kinase	26.77/8.24	26.6/5.29	103	16.26	1.57	0.61	0.4
47	GI:224109060	Phosphoglycerate kinase	50.21/8.25	59.38/6.36	104	9.36	2.14	2.09	0.87
84	GI:566185071	Mitogen-activated protein kinase 6	64.12/8.79	65.44/6.43	105	11.21	1.24	1.53	1.66
85	GI:224130362	Mitogen-activated protein kinase 3	42.75/5.76	40.14/5.46	267	12.63	1	1.58	2.01
46	GI:224143653	Hexokinase 1	53.09/5.65	62.15/6.36	103	11.04	1.77	1.85	0.97
25	GI:224081987	Transcription factor bHLH	40.43/5.09	45.79/4.51	169	12.6	1.65	1.73	0.88
11	GI:566205899	homeobox-leucine zipper protein	77.74/6.33	76.23/4.93	173	8.77	0.85	0.81	2.23
50	GI:224131376	Heat shock transcription factor	50.78/5.82	59.9/6.63	142	10.16	1.54	1.03	0.52
Predicted protein									
7	GI:566150698	Hypothetical protein	88.89/5.45	85.03/5.27	123	8.31	1.94	2.37	1.75
76	GI:566185140	Hypothetical protein	20.87/9.55	20.61/5.63	97	17.35	0.84	0.38	0.26
83	GI:566201677	Hypothetical protein	128.15/5.82	129.14/6.36	105	6.76	6.99	2.84	2.58

^a^, Database accession numbers according to NCBInr.

^b^, Experimental Mw/pI.

^c^, Theoretical Mw/pI.

^d^, The Mascot search score against the database of NCBInr.

^e^, Sequences coverage.

^f^, protein spots showed a significant change in abundance (fold change) by a factor>1.5-fold compared to the control analyzed.

Eighty-three differentially expressed proteins were isolated and detected by MALDI-TOF/TOF-MS analysis and identified by screening against the NCBI nr protein database (Table 1 and S1 Table). The proteins were classified into nine functional categories: response to stress (26%), material metabolite (21%), photosynthesis and energy metabolite (14%), DNA and ion binding (13%), antioxidant activity (13%), protein kinase (8%), and predicted protein (4%) (Fig 3c). Within each of the GO categories, the dominant subcategory was “response to stress”, and included heat shock proteins (spots 64, 74, 10, 12, 14 and 72), a 14-3-3 protein (spot 58), CCoAOMT (spots 67 and 62) and NBS-LRR resistance gene-like protein (spot 3). These results demonstrated that processes involving all of these proteins were important in the response to Cd stress in *P*. *yunnanensis*. Additionally, antioxidant proteins (spots 1, 48, 69, 56, 61, 22 and 57), protein kinases and transcription factors (spots 74, 84, 85 and 25) accounted for nearly 22% of all differentially-expressed proteins (Table 1 and Fig 3c).

To identify key metabolic processes that were affected by Cd, Eighty-three proteins that were identified were further mapped in MapMan. The result revealed that most of the Cd-responsive proteins (27 proteins) involved in biotic stress signaling were activated (Fig 3d, S2 Fig and S2 Table). In the “response to stress” category, heat shock proteins (HSPs), as well as proteins implicated in cell wall metabolism such as CCoAOMT (spots 67 and 62), were highly overexpressed (Fig 3d and Table 1). Several protein kinases involved in biotic stress, such as MPK3 and MPK6 (spots 84 and 85), were also activated (Fig 3d and Table 1). These data suggest that Cd induced responses similar to the hypersensitive response upon plant-pathogen interaction and that these responses may have also invoked the activation of cell wall metabolism.

### Changes in ROS and MDA contents

**Fig 4:**
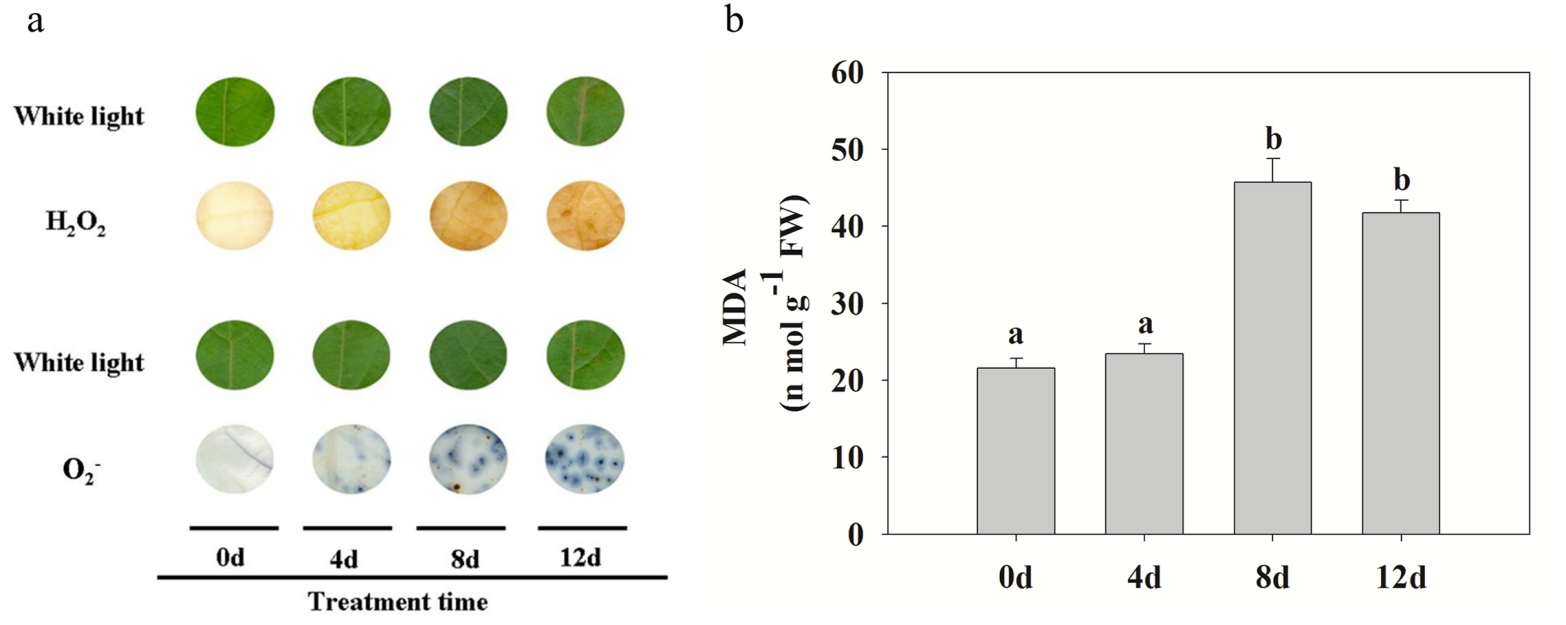
Effect of Cd stress on ROS accumulation and MDA in poplar leaves. (a) *In situ* detection of changes in leaf ROS (H_2_O_2_ and O_2_^−^) levels at different times under Cd treatment. Images were obtained using a scanner at the indicated times. Images captured under white light were used as controls. The strong or weak of brown (H_2_O_2_) and blue (O_2_^−^) reflects the accumulation level of ROS products. (b) MDA content at different times under Cd treatment. Data represent the means of three replicate experiments (*n* = 30/experiment). Means labeled with different letters are significantly different according to Tukey’s test (P < 0.05). The raw data are provided in S3 Table.

To determine whether CdCl_2_ induced H_2_O_2_ accumulation, the levels of H_2_O_2_ and MDA in *P*. *yunnanensis* leaves were determined. Compared with the control, H_2_O_2_ and O_2_^−^, which reflect levels of cellular oxidation, both gradually accumulated when plants were exposed to Cd stress treatment (Fig 4a). MDA, as an end product of lipid peroxidation, was also increased by the Cd treatment. No significant differences were recorded in the MDA levels of *P*. *yunnanensis* from 0 to 4 d of treatment, but as shown in Fig 4b, MDA levels increased rapidly from 4 to 8 d of treatment.

### Changes in antioxidant enzyme activities

**Fig 5:**
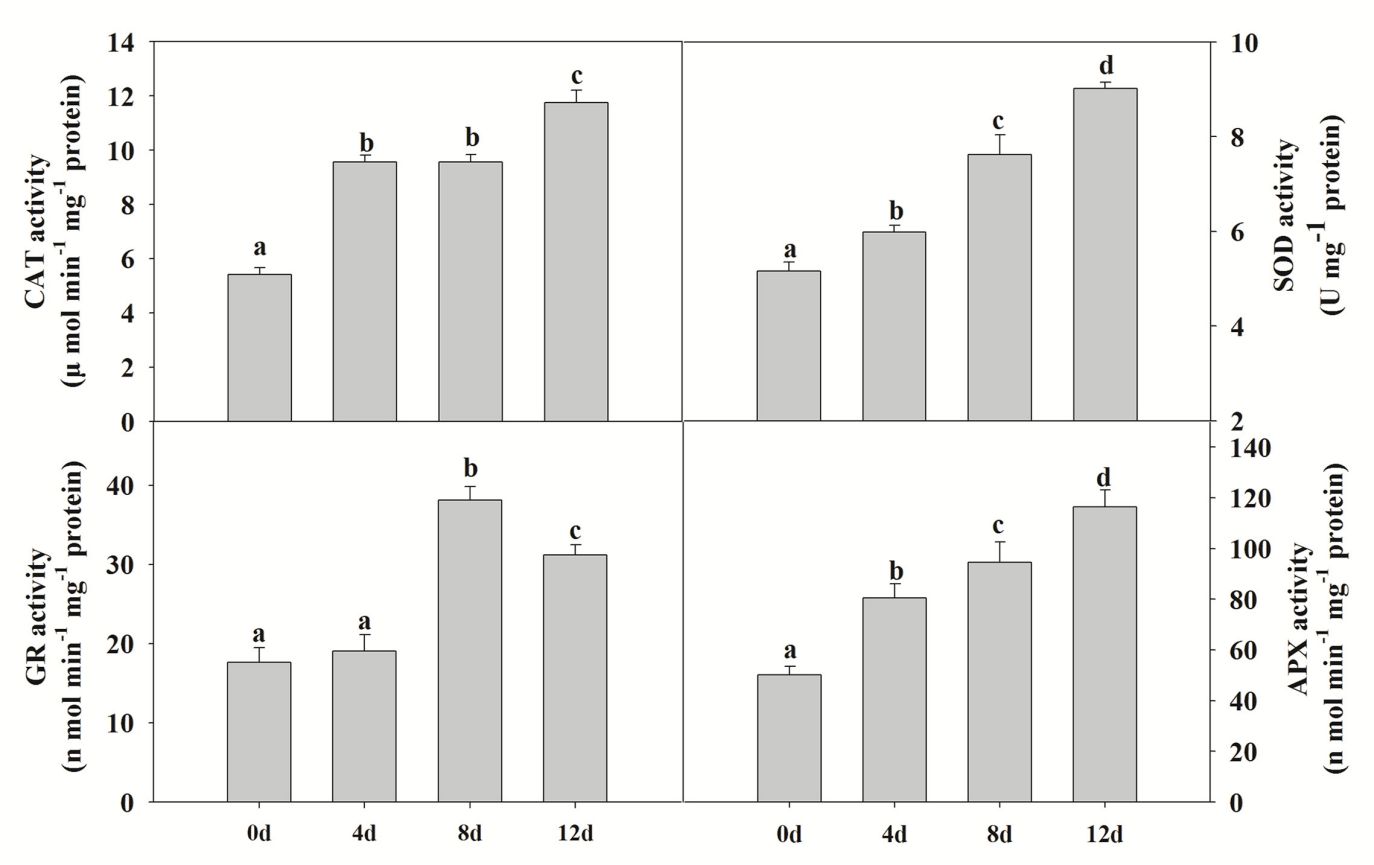
The effects of cadmium on antioxidant enzyme activities in poplar (CAT, APX, SOD and GR). Plants were treated with cadmium as above, and the antioxidant enzyme activities were determined using colorimetric methods. Values reflect means ± SEs of at three independent experiments (*n* = 30/experiment). Different symbols above the bars indicate significant differences (Tukey’s test, *P* < 0.05). The raw data are provided in S4 Table.

Environmental stress inhibits the growth and photosynthetic abilities of plants because of the breakdown of the balance between antioxidant defense and ROS production, which can lead to damage of proteins, membrane lipids and other cellular components [28]. The proteomic results (above) indicated that increasing Cd stress promotes the accumulation of antioxidant enzymes such as APX and CAT (Table 1, spots 57 and 48), and thus the capacity to reduce toxic levels of ROS. Thus, we measured the activities of CAT, APX, GR and SOD in leaves of *P*. *yunnanensis*. Significant increases in APX, CAT and SOD activities were observed with increasing Cd stress duration (Fig 5). GR activities increased markedly from 4 to 8d stress, and then decreased slightly after 12 d stress (Fig 5).

### Western blot analysis of up-regulated proteins

**Fig 6:**
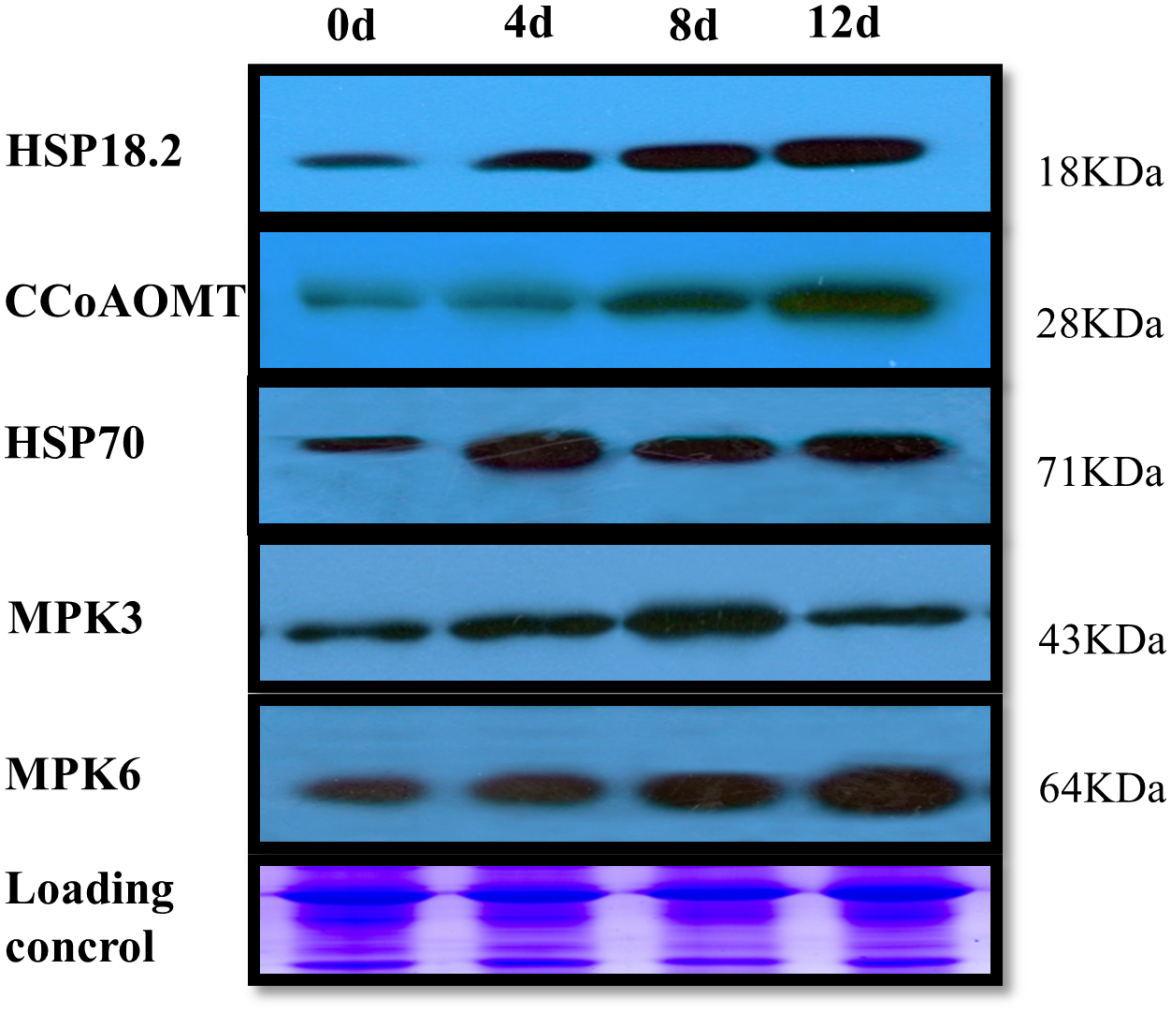
Western blotting analysis of proteins from poplar leaves. Total protein samples were separated by SDS-PAGE and electroblotted onto a PVDF membrane. The part of equal amounts of protein downloading SDS gels was stained with Coomassie Blue as a loading control.

The proteomic analysis also revealed that defense-related protein and kinase levels increased in response to Cd stress. Therefore, we performed western blot analysis with specific antibodies against plant MAPK6, MAPK3, HSP70, HSP18.2, and CCoAOMT (Fig 6 and S3 Fig). Accumulation of these five proteins was induced to varying degrees by Cd stress treatment. Similar to the results of the proteomic analysis, these proteins showed different expression levels. MAPK6, HSP18.2 and CCoAOMT were induced rapidly from 0 to 12 d (Fig 6). The expression peaks of HSP70 and MAPK3 occurred at 4 and 8 d, respectively, after the start of the treatment (Fig 6).

## Discussion

### Photosynthesis and energy-associated protein changes during Cd stress

In plants, photosynthetic capabilities are mainly regulated via photochemical reactions facilitating energy production, gas exchange and CO_2_ fixation and assimilation. Cd damages photosynthetic capabilities, induces oxidative stress, inhibits stomatal opening and reduces the absorption of nitrates and iron [13,29]. In the present study, Cd caused a decrease in the photosynthetic activity of *P*. *yunnanensis* (Fig 1). Cd ions can affect photosynthesis via inhibiting the activity of RuBisCO and damaging its structure by substituting for Mg ions. Moreover, Cd caused an irreversible dissociation of the large and small subunits of RuBisCO, thus also leading to total inhibition of the enzyme [13]. However, some proteins associated with protection and repair mechanisms related to the photochemical reaction pathway were differentially expressed, such as three RuBisCO proteins (Fig 2a, spots 49, 68 and 79) and glyceraldehyde 3-phosphate dehydrogenase (GADPH) (Fig 2a, spot 43), which were up-regulated in *P*. *yunnanensis* leaves during Cd treatment, with expression peaks at 4 and 8 d, respectively (Fig 2a and Table 1). Other authors observed similar results in Cd-treated poplar leaves: RuBisCO and binding proteins showed a decrease in abundance under longer-term (56 d) Cd stress [30]. RuBisCO for carboxylation and GADPH for carbon reduction are key enzymes for maintaining higher photosynthetic capacity in plant responses to environmental stresses [31]. In *P*. *yunnanensis*, the accumulation of RuBisCO and GADPH suggests a greater photosynthetic CO_2_ fixation capability during the early stages, and then decreased during the later stages of Cd stress. Chlorophyll a-b binding protein and chlorophylls *a* and *b* constitute the light-harvesting complex (LHC). The LHC functions as a light receptor that captures and delivers excitation energy to photosystems I and II, with which it is closely associated [32]. In the present study, the increase of chlorophyll a-b binding protein 2 (Fig 2a, spot 63) after 4 d of Cd stress suggests that this protein provides energy for the photosystems. Our results clearly demonstrate that *P*. *yunnanensis* used multiple mechanisms to enhance its photosynthetic activity in response to Cd damage until the Cd stress exceeded plant tolerance limits (after 4 d).

### Stress-responsive protein changes during Cd stress

Stress-responsive proteins, including heat shock protein (HSP) family members (HSP22, spot 64; HSP20, spot 74; HSP90, spot 10; HSP23.1, spot 72; HSP70, spot 14), a 14-3-3 protein (spot 58) and CCoAOMT (spots 67 and 62), were increased in *P*. *yunnanensis* following exposure to Cd treatment. Heat-shock proteins play a crucial role in protecting plants against stress by re-establishing normal protein conformations and thus cellular homeostasis. HSP20 (Fig 2, spot 74), HSP70 (Fig 2, spot 14) and HSP90 (Fig 2a, spot 10) were increased in *P*. *yunnanensis* leaves under Cd stress, as confirmed by western blotting (Fig 6). HSP20, HSP70 and HSP90 have a similar function, maintaining the functional conformations of proteins and preventing the aggregation of non-native proteins, and can assist in protein refolding under stress conditions [33,34]. For instance, the correct folding of RuBisCO requires the HSP70 chaperone and a more efficient RuBisCO could potentially reduce photosynthetic water use, increasing plant tolerance to drought stress [35]. The increase in HSP70 may explain the enhanced RuBisCO content of *P*. *yunnanensis* under Cd stress conditions by helping with correct folding to maintain the photosynthetic capability of *P*. *yunnanensis* leaves.

In plant cells, 14-3-3 proteins have been widely implicated in various physiological processes, such as stress responses, signal transduction, metabolism, cell growth and development [36,37]. Aluminum (Al) stress enhanced the expression of 14-3-3 proteins to maintain a high level of plasma membrane H+-ATPase activity in Al-tolerant soybean roots. However, Al stress reduced the expression levels of 14-3-3 proteins and their binding to phosphorylated plasma membrane H+-ATPase in Al-sensitive soybean roots. The lack of protection through 14-3-3 protein binding led to unstable phosphorylation of PM H+-ATPase in Al-sensitive soybeans under Al stress [38]. In the present study, a 14-3-3 protein (Fig 2a, spot 58) and H+-ATPase (Fig 2a, spots 37 and 38) were up-regulated substantially following Cd treatment, supporting the possibility of cross-talk between 14-3-3 proteins and H+-ATPase during Cd exposure.

The cell wall acts as a mechanical protection against environmental stresses [39]. Research on the subcellular deposition and localization of heavy metals has shown that most Cd is localized in cell walls, and the cell wall is one of the major storage sites for Cd in the cell [40,41]. Liu *et al*. indicated that metal-tolerant plants can store more Cd in their cell walls than metal-sensitive plants [42]. Our proteomics results showed that two CCoAOMT proteins (Fig 2a, spots 67 and 62) had higher expression levels after 4 and 12 d of stress (Fig 2a and Table 1). Higher expression of CCoAOMT can regulate the synthesis of plant cell walls [43]. Drought stress induced the expression of CCoAOMT in rice, which enhanced lignification and increased resistance to drought stress [44]. Expression of CCoAOMT was also induced by drought stress in maize leaves, tobacco, maritime pine and *Arabidopsis* roots [45–47]. In *P*. *yunnanensis*, CCoAOMTs were up-regulated, suggesting that these proteins function to promote cell wall synthesis for greater Cd storage to reduce intracellular damage in response to Cd exposure.

### Antioxidant enzyme and related protein changes during Cd stress

Antioxidant enzymes maintain the cellular redox status at an acceptable level to avoid damage from the over-accumulation of ROS [48,49]. ROS can be produced by different pathways such as imbalance of the electron transport chains in both chloroplasts and mitochondria [50]. Thus, Cd could lead to the generation of ROS by production of a disturbance in the chloroplasts. In our proteomic analysis, we detected differential expression of seven antioxidant proteins. Peroxiredoxin (spot 61) can reduce hydrogen peroxide and alkyl hydroperoxides using reducing equivalents provided through the thioredoxin or glutaredoxin system. It is up-regulated by drought-induced oxidative stress and decreases H_2_O_2_ levels in rice chloroplasts [51] and, as an electron acceptor in CDSP32-driven electron-transfer, protects the photosynthetic apparatus from oxidative damage in *Arabidopsis* [52]. CAT and APX, two major ROS-scavenging enzymes in plants, provide cells with a highly efficient system for removing superoxide and hydrogen peroxide. CAT and APX can catalyze the decomposition of H_2_O_2_ to H_2_O [53]. Antioxidant-detoxifying proteins showed an increased abundance such as peroxidases and aldehyde dehydrogenases, as well as quinone reductases in poplar leaves after Cd stress [22]. Here, we measured the activities of enzymes including CAT, APX, SOD and GR, and the production of H_2_O_2_ in *P*. *yunnanensis* to further investigate the relationship between H_2_O_2_ and antioxidant enzymes. We found that the activities of these enzymes and the production of H_2_O_2_ and O_2_^−^ were increased by Cd treatment. Cd can interfere with the antioxidant system, and indirectly produce ROS which cause oxidative damage to plants. The MDA content of *P*. *yunnanensis* leaves gradually rose over the course of the Cd stress treatment, with the greatest increase recorded after 8 d stress (Fig 4b). Thus, our results indicate that Cd stress changes the original balance in the antioxidative metabolism of *P*. *yunnanensis* plants as well as increasing ROS production, and induces the expression of antioxidant proteins as an adaptive response to neutralize excess ROS and minimize damage during the early stages (0–4 d). However, severe oxidative damage occurred along with obvious phenotypic changes during the later stages (8–12 d).

### Proteins kinase and transcription factor changes during Cd stress

MPK3 and MPK6 are two members of the TEY subtype MAPKs. Recently, it was proven that MPK3 and MPK6 are activated by Cd-induced ROS accumulation in *Arabidopsis* [54]. Research has shown that MPK3 and MPK6 are involved in responses to many biotic and abiotic stresses such as wounding, pathogen, ABA, cold, salt, osmotic and oxidative stresses [55]. Heavy metal-induced MAPK signaling has been investigated in alfalfa and rice [56,57]. Our proteomic results demonstrate that two MAPKs, MPK3 and MPK6, were differentially regulated. Additionally, Roelofs *et al*. speculated that downstream targets of MAPK signaling could be bZIP, MYB and MYC transcription factors during plant metal stress after comparing known signaling pathways induced by metals stress and other abiotic stresses between soil invertebrates and plants [58]. We found much higher levels of a Cd-induced homeobox-leucine zipper protein (spot 11) in *P*. *yunnanensis* after 12 d stress (Fig 2a). However, it is unknown whether this homeobox-leucine zipper protein would be regulated by the MPK3 and MPK6 identified in our study. Transcriptional control of the expression of stress-responsive genes is a crucial part of plant responses to abiotic and biotic stresses [59]. A heat shock transcription factor (spot 50) increased after the earlier stage of Cd exposure in the present study, which is consistent with the finding that Cd stress increases the expression levels of heat shock proteins. Taken together, the upregulation of these protein kinases and transcription factors suggests that these proteins function to improve the tolerance of *P*. *yunnanensis* to Cd stress.

## Conclusions

**Fig 7:**
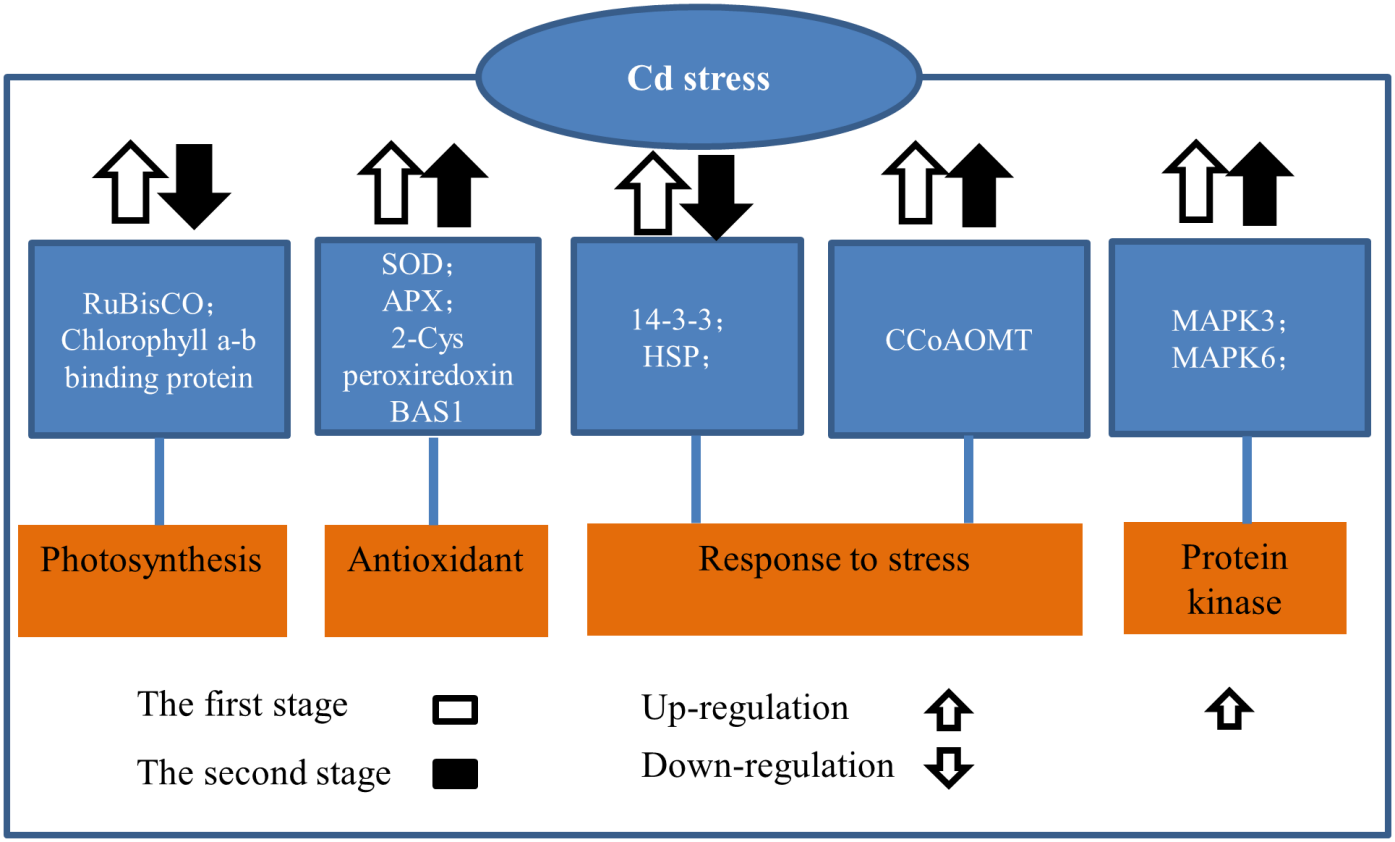
Proposed model demonstrating that poplar applies multiple strategies in response to Cd conditions.

Our physiological and proteomic profiling of poplar (*P*. *yunnanensis*) has provided an insight into how woody plants respond to excessive Cd stress. We detected two stages in the response to Cd stress, and propose a model to explain the poplar response to Cd stress based on our results (Fig 7). During the first stage, transiently induced defense-response molecules, photosynthesis- and energy-associated proteins, antioxidant enzymes and HSPs accumulate to enhance protein stability and establish a new cellular homeostasis. This activity explains why plant photosynthetic capability during this period barely changed. During the second stage, a decline in RuBisCO and HSP levels leads to an imbalance of the plant photosynthetic system. Antioxidant enzyme activities increased seem not be able to entirely counteract Cd-induced ROS overproduction in plants. However, higher expression of CCoAOMT may regulate plant cell wall synthesis for greater Cd storage. Meanwhile, the expression of MPK3, MPK6 and a homeobox-leucine zipper protein was higher in the second stage. These genes may be candidates for further research and use in genetic manipulation of poplar tolerance to Cd stress.

## Supporting Information

S1 Fig 2-DE gel maps following cadmium treatment. The 2-DE gel of total proteins from leaves treated with 100 μM Cd for 0 d, 4 d, 8d and 12 d, repectively. A: Control (0 d); B: after 100M Cd for 4 d; C: after 100 μM Cd for 8 d; D: after 100 μM Cd for 12 d. Those that changed significantly in response to Cd are indicated by red arrows. R1, R2 and R3 mean three replicates.
(TIF)

S2 Fig Proteins involved in stress according to MapMan in leaves after 0d, 4d, 8d and 12d of treatment. (TIF)

S3 Fig Western blotting analysis of proteins from poplar leaves of different treatments. R1, R2 and R3 mean three replicates. The part of equal amounts of protein downloading SDS gels was stained with Coomassie Blue as a loading control.
(TIF)

S1 Table Identification of differentially expressed proteins in poplar after Cd treatment. (XLSX)

S2 Table Proteins involved in biotic tress signaling according to MapMan in leaves after 0d, 4d, 8d and 12d of treatment. (XLSX)

S3 Table The raw data for MDA detection of *P*. *yunnanensis* after Cd treatment. (XLSX)

S4 Table The raw data for CAT, APX, SOD and GR detection of *P*. *yunnanensis* after Cd treatment. (XLSX)
