# Supplementary figures and images for: Comparative Physiological and Proteomic Analysis Reveals the Leaf Response to Cadmium-Induced Stress in Poplar (Populus yunnanensis)
Source: PLoS One. 2015 Sep 8;10(9):e0137396. doi: 10.1371/journal.pone.0137396 (PMC4562643; doi:10.1371/journal.pone.0137396)

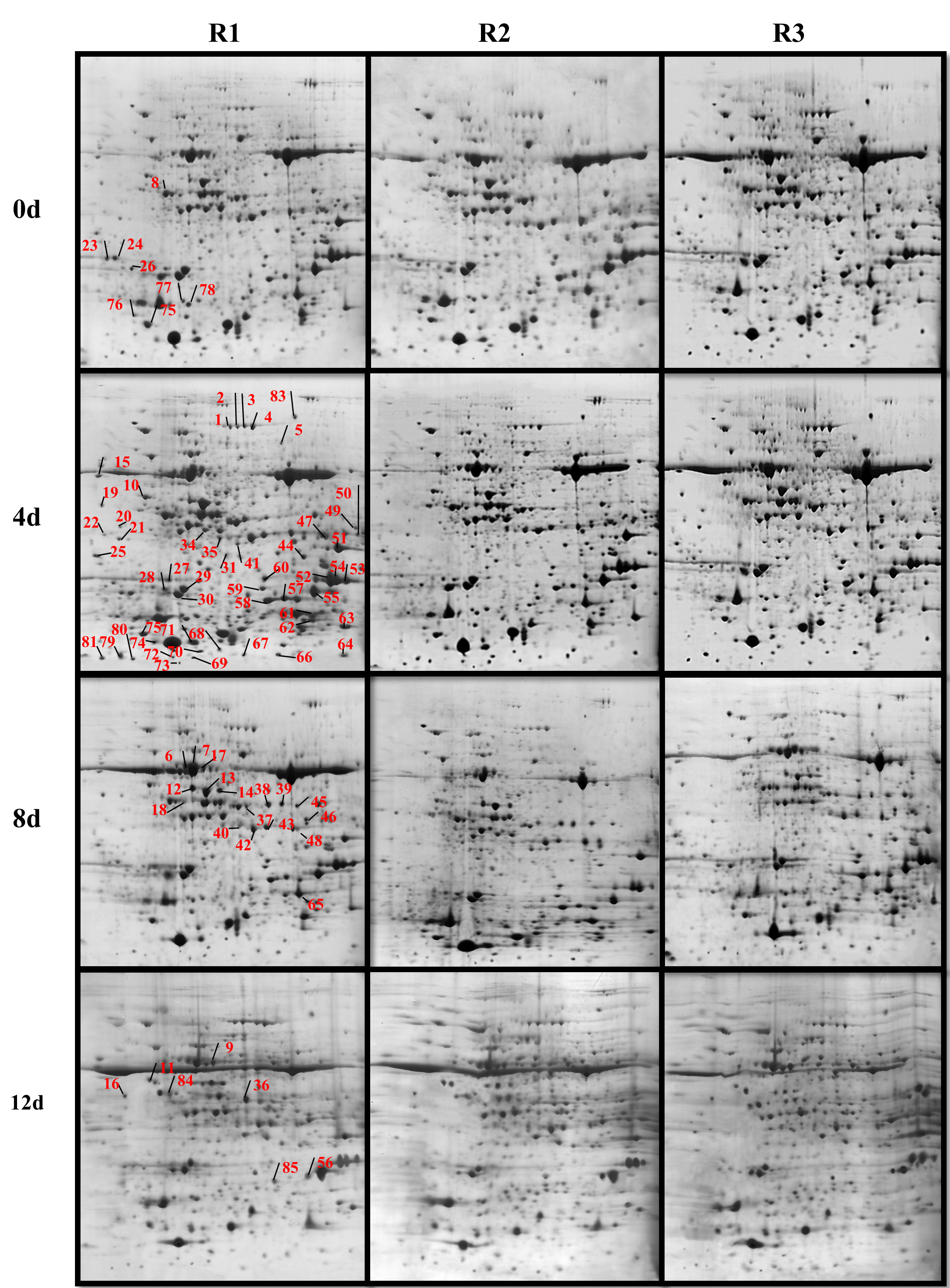

Supplement: S1 Fig — The 2-DE gel of total proteins from leaves treated with 100 μM Cd for 0 d, 4 d, 8d and 12 d, repectively. A: Control (0 d); B: after 100M Cd for 4 d; C: after 100 μM Cd for 8 d; D: after 100 μM Cd for 12 d. Those that changed significantly in response to Cd are indicated by red arrows. R1, R2 and R3 mean three replicates. (TIF) [file pone.0137396.s001.tif]

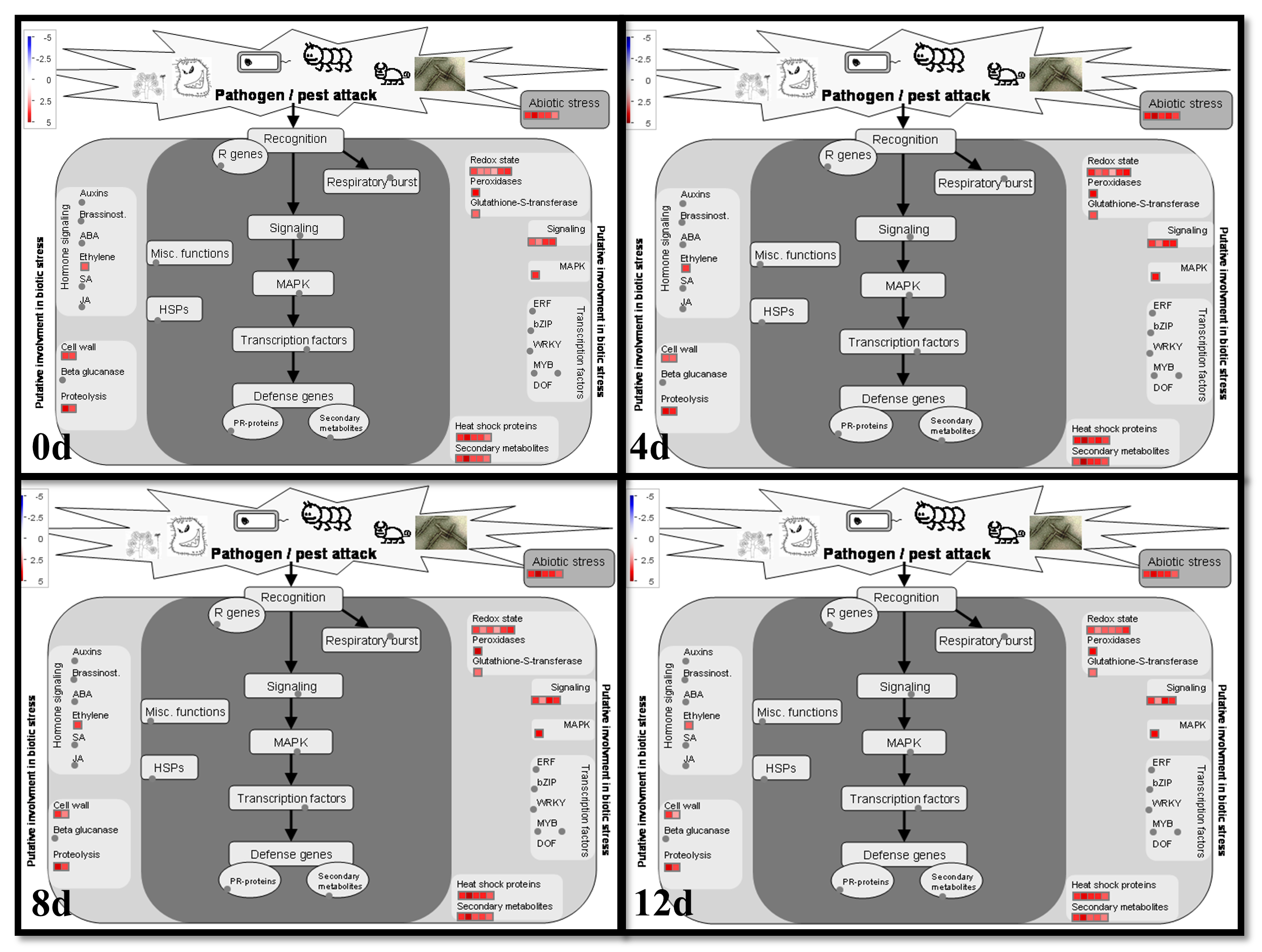

Supplement: S2 Fig — (TIF) [file pone.0137396.s002.tif]

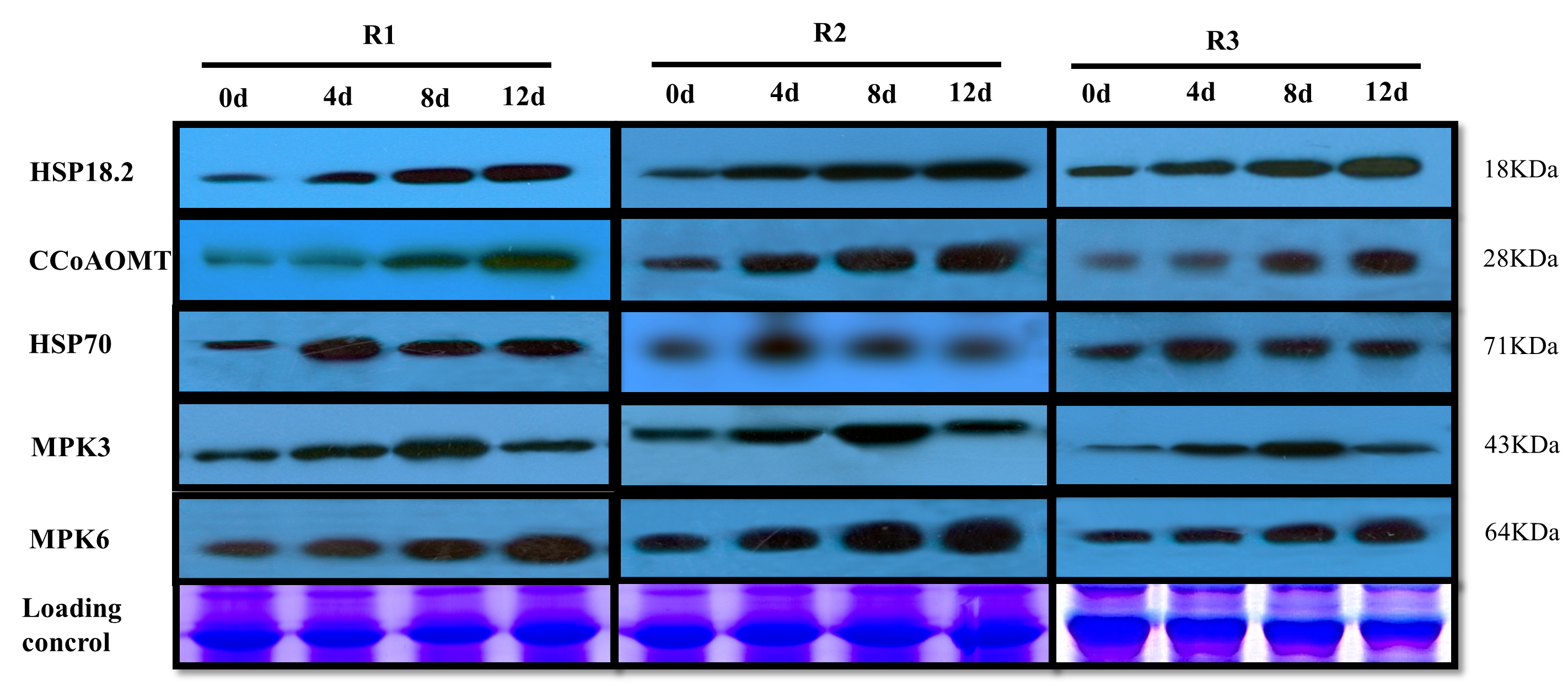

Supplement: S3 Fig — R1, R2 and R3 mean three replicates. The part of equal amounts of protein downloading SDS gels was stained with Coomassie Blue as a loading control. (TIF) [file pone.0137396.s003.tif]
